# FEM Analysis of Various Multilayer Structures for CMOS Compatible Wearable Acousto-Optic Devices

**DOI:** 10.3390/s21237863

**Published:** 2021-11-26

**Authors:** Mehwish Hanif, Varun Jeoti, Mohamad Radzi Ahmad, Muhammad Zubair Aslam, Saima Qureshi, Goran Stojanovic

**Affiliations:** 1 Department of Electrical and Electronics Engineering, Universiti Teknologi PETRONAS, Seri Iskandar 32610, Perak, Malaysia; mohamadradzi.ahmad@utp.edu.my (M.R.A.); m.zubair_g02974@utp.edu.my (M.Z.A.); 2 Faculty of Technical Sciences, University of Novi Sad, Trg Dositeja Obradovica 6, 21000 Novi Sad, Serbia; varunjeoti@uns.ac.rs (V.J.); saima.qureshi@uns.ac.rs (S.Q.); sgoran@uns.ac.rs (G.S.)

**Keywords:** figure of merit, acousto-optic, SAW, LiNbO_3_, ZnO, AlN, SiO_2_, multilayer structures, piezoelectric, optics, COMSOL, FEM

## Abstract

Lately, wearable applications featuring photonic on-chip sensors are on the rise. Among many ways of controlling and/or modulating, the acousto-optic technique is seen to be a popular technique. This paper undertakes the study of different multilayer structures that can be fabricated for realizing an acousto-optic device, the objective being to obtain a high acousto-optic figure of merit (AOFM). By varying the thicknesses of the layers of these materials, several properties are discussed. The study shows that the multilayer thin film structure-based devices can give a high value of electromechanical coupling coefficient (*k*^2^) and a high AOFM as compared to the bulk piezoelectric/optical materials. The study is conducted to find the optimal normalised thickness of the multilayer structures with a material possessing the best optical and piezoelectric properties for fabricating acousto-optic devices. Based on simulations and studies of SAW propagation characteristics such as the electromechanical coupling coefficient (*k*^2^) and phase velocity (*v*), the acousto-optic figure of merit is calculated. The maximum value of the acousto-optic figure of merit achieved is higher than the AOFM of all the individual materials used in these layer structures. The suggested SAW device has potential application in wearable and small footprint acousto-optic devices and gives better results than those made with bulk piezoelectric materials.

## 1. Introduction

In the last few decades, photonic on-chip sensors have gained significant attention amongst the various attractive photonic integrated circuits (PICs) applications [1,2,3,4]. Moreover, due to the advancement in optic structures (gratings, waveguides, etc.), photonics-based sensors have also gained considerable interest in several other fields like telecommunication and bio-medical instrumentation [5]. In the area of integrated optics, surface acousto-optic (AO) devices such as tunable optical filters, modulators, and optical switches are extensively used [6,7,8]. As the demand for AO devices increases, the interest in using AO material with high sensitivity towards acoustic or optic stimulants also increases.

**Figure 1:**
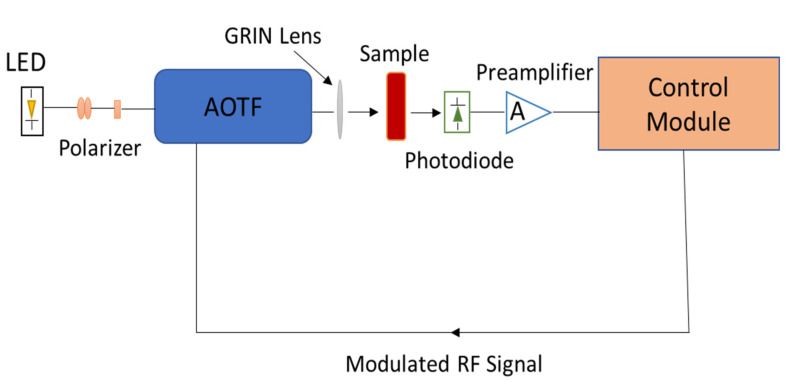
A schematic of the experimental setup of an AOTF-based spectrometer.

The application of acousto-optic tunable filters (AOTFs) in spectroscopy has been well recognised in the past several years. Spectrometers based on AOTF technology have several appealing design characteristics. Among these advantages, the most prominent ones are high spectral resolution, less rf driving power, high durability, compact size, maintenance-free functioning, lightweight and quick data gathering [9]. Another important feature of AOTFs is, that they can simultaneously and individually filter a wide range of optical wavelengths by operating the acoustic transducer at various corresponding frequencies. They are thus suited for use in spectrometer instruments as the tuning element. A schematic of a typical miniaturized AOTF spectrometer is shown in Figure 1. In the past few years, efforts have been made to incorporate spectrometers in wearable sensors. AOTF-based spectrometers possess the potential to be incorporated into such sensors [10]. However, to design such a device the features and design parameters (acousto-optic) of an AOTF must be taken into consideration. As they play a vital role in the miniaturization of the device [11].

Previously, acousto-optic parameters were primarily investigated in the domain of crystallography; however, the focus has now switched to device optimisation [12]. Most of the photonics-based sensors work on the principle of acousto-optic effect in which there is a surface acoustic wave (SAW) device that acts as piezoelectric and optic devices. The most attractive features of SAW devices are that they consume less power and have a fast response time. So far, typical piezoelectric materials utilised in acousto-optic devices are langasite (La_3_Ga_5_SiO_14_), lead molybdate (PbMoO_4_), quartz (SiO_2_), tellurium dioxide (TeO_2_), lithium tantalite (LiTaO_3_),) and lithium niobate (LiNbO_3_) [4]. These bulk piezoelectric materials are used in most acoustic devices, but they are generally expensive and difficult to integrate with electronics, and some are even toxic in nature. Moreover, they are usually delicate and fragile if polished into extremely thin structures for high-frequency applications. In addition to this, using these large bulk materials is impractical in terms of cost, size, acousto-optic figure of merit, and integration [13]. The optimum thickness of the substrate required for SAW applications is normally around 10 λ or more. This thickness is chosen so that all the SAW travel on the top surface (piezoelectric material) and eventually dies out when it travels deeper into the substrate. However, for acousto-optic purposes where SAW interacts with light over an optical waveguide, the waveguide thickness can be of the order of 1 or more wavelengths of the infrared maximum range—nearly 5 microns or less. Hence, the multilayer substrate thickness is expected to be in excess of 10 s of the wavelength while the optical waveguide thickness can be about 1 wavelength or so. Attempts to replace these bulk materials with thin films have produced promising results [14]. Here, we emphasise the need to evaluate and optimise these thin film structures for achieving a better acousto-optic figure of merit.

## 2. Literature Review

The use of thin film-based optical devices [15,16,17,18] has increased recently due to the advancement of thin-film deposition technology that can produce thin films with high uniformity, homogeneity, etc. These devices typically use single-layer thin films and can achieve remarkable performance. However, the performance of these devices can be further improved by considering alternative thin films that have been made available recently [19]. Using these thin films in a multilayer structure can significantly enhance the device’s performance. For instance, in designing optical filters or SAW devices, multilayer structures improve phase velocity, temperature stability, and mechanical strength [20,21,22]. In addition to this, the use of multilayer thin films is also preferable for broad tunability compared to the doped semiconductors, as these multilayer thin films give the desired response for various targeted applications. A typical example of it is an acousto-optic tunable filter (AOTF) [23,24,25,26]. The performance of devices utilising multilayered structures is dependent on various properties of a material medium such as acoustic, optic, and acousto-optic properties using both the sound and light wave interaction. For such devices to significantly improve the productivity of acousto-optic interaction, strong SAW interaction and optical waveguiding with low loss are essential. Thus, the determination of a reasonable layered structure is necessary. The layered structure utilised in most of the acousto-optic devices ought to have the characteristics such as high electromechanical coupling coefficient (*k*^2^) and phase velocity (*v*) [27]. These parameters are then formulated into the acousto-optic figure of merit that can measure the suitability of such materials for AO devices.

The acousto-optic figure of merit measures a material’s capacity to modulate diffraction intensity. For the effective realisation of such devices, it is better to make the blend of layer structure by utilising the materials having said properties [7,19]. Zinc oxide (ZnO) thin films exhibit good piezoelectric characteristics with high *k*^2^, sensitivity, reliability and attractive optical properties. [28,29]. On account of promising optical, electro-optic, piezoelectric, photoelastic, photorefractive and elastic properties [30], ferroelectric lithium niobate (LiNbO_3_) is also broadly utilised in guided-wave optics. Notwithstanding that, another component tends to be developed on various substrates, incorporating silicon (Si). Additionally, aluminium nitride (AlN)-based layered SAW has attracted significant interest [25,26,27,28]. It is mainly popular among other piezoelectric materials because of its high acoustic velocity (5760 ms^−1^). Another important feature of AlN thin film is its ease of integration into complementary metal-oxide-semiconductor (CMOS) platforms when contrasted with other piezoelectric materials like LiNbO_3,_ ZnO, LiTaO_3_ and quartz, as these materials create contamination in integrated circuit (IC) processes [31,32]. Within the last several years, there has been a tremendous rate of increase in interest in CMOS compatible surface acoustic wave (SAW) devices. The main reason to prefer CMOS compatible SAW devices over other devices is to eliminate the parasitic capacitance effect (mainly occurs when discrete SAW devices are integrated circuit connections are bonded). Furthermore, another reason is to minimise the footprints by merging the SAW device and the integrated circuit on a single chip.

In this manuscript, we investigated three multilayered structures A (ZnO/SiO_2_/Si), B (LiNbO_3_/SiO_2_/Si), and C (AlN/SiO_2_/Si). There are three piezoelectric materials used in this study (i.e., AlN, ZnO, and LiNbO_3_). AlN and ZnO are in c-axis orientation with texture 11$\overset{\_}{20}$ and for LiNbO_3_, 128° Y-cut Z-propagating is used. The top layers, i.e., LiNbO_3_, AlN, ZnO, are commonly used as bulk materials in AO devices with good piezoelectric and optic properties [20,21,22,23]. By comparing the propagation properties of SAW within said structures, the acousto-optic figure of merit of these three multilayer structures is then calculated.

## 3. Materials and Methods

This work uses the COMSOL Multiphysics software to perform 2D finite element method (FEM) simulations (modal and harmonic analysis) on the layered SAW device. Researchers used, validated, and reported on the results of the FEM study on different piezoelectric materials [33,34,35,36]. The complexity of modelling the SAW modes with longitudinal and shear vertical particle displacements can be significantly reduced using 2D structures. Furthermore, the use of an infinite set of IDTs helps in simulating a standing wave pattern that results when a resonator is employed. So, a unit cell with periodic boundary conditions on either side of the cell becomes a useful tool to simulate a SAW resonator with an infinite set of IDTs [32,37,38,39,40].

**Figure 2:**
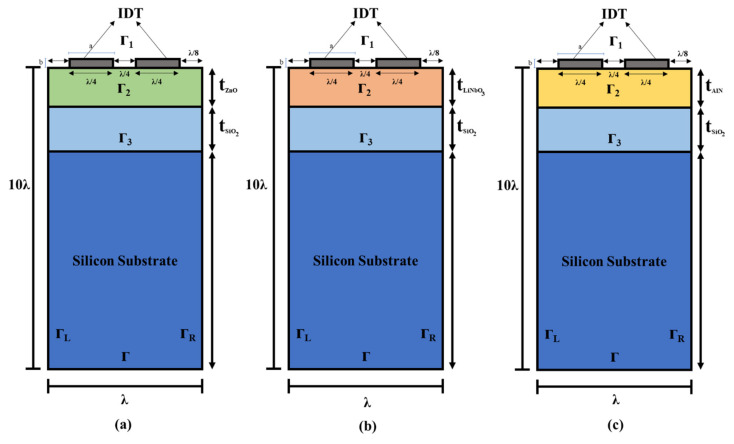
A two-dimensional unit cell geometry used in FEM simulation for the multilayer structure (**a**) (ZnO/SiO_2_/Si), (**b**) (LiNbO_3_/SiO_2_/Si) and (**c**) (AlN/SiO_2_/Si).

To determine the acousto-optic figure of merit, various SAW propagation characteristics, such as phase velocity and *k*^2^, are calculated and used. The electromechanical coupling coefficient (*k*^2^) is a numerical measure of the conversion efficiency between electrical and acoustic energy in piezoelectric materials. In this study, all three structures work on the principle of the acousto-optic effect in which there is a surface acoustic wave (SAW) device that acts as a piezoelectric and optic device. In designing an efficient SAW-based acousto-optic device, one of the concerns is the driving power. The input driving power is directly related to the electromechanical coupling coefficient. Higher the *k*^2^, lesser is the input power needed to convert the electrical energy to acoustic energy, which, as a result, enhances the device efficiency. Hence, the electromechanical coupling coefficient is the parameter that enables us to know the efficiency of the acousto-optic device. Additionally, the electromechanical coupling coefficient, which is a parameter that also depends on the piezoelectric film thickness, is also related to acoustic bandwidth. Higher bandwidth allows for a wider range of modulation for RF carrier, which, in turn, means a wider range of tunability of acousto-optic filters. Thus, this study analyses the FEM 2D models A, B, and C (Figure 2). Table 1 summarises the dimensions of the SAW device used in the simulation, while Figure 2 details the geometry of the 2D single-cell multilayered SAW device.

Hence, for simulating a SAW device, a single cell measuring one λ is taken. A total of 10 λ is taken as the depth of a unit cell because SAW is generated on the top surface and dies out in the lower boundary as it travels down into the substrate. The material used for IDTs in this device is aluminium (Al). The properties of Al are obtained from the inbuilt COMSOL library, which is the density of 2700 kilogrammes per cubic metre, 70 GPa Young’s modulus, and a Poisson’s ratio of 0.33. The ratio of metallisation is taken as 50% (2a/λ), while 2.5% is the electrode’s relative thickness (b/λ). Here, the width and height of electrodes are a (λ/4) and b (100 nm), respectively. To improve the accuracy of the results, a very thin triangular mesh is used for the simulations. Both the electrodes, from the top and sides, are free in terms of mechanical boundaries. The boundaries of the left electrode are grounded, and for the right one, it is set to a floating potential with no charge accumulation on the surface. The boundary conditions utilised in the simulations are shown in Table 2, and the material constants used in the simulations are listed in Table 3. All the material constants mentioned in Table 3 are taken from reference [41,42,43,44].

In general, the equation for determining the acoustic wave velocity is as follows:

(1) v=fλ

The central frequency is denoted by *f*, and the wavelength of the acoustic wave is represented by *λ*. To find the velocity in eigenmode, the following equation is used:

(2) $$v=\left(f_{res}+f_{anti}\right)p$$

Here, both $f_{res}$ and $f_{anti}$ frequencies denote the resonant and anti-resonant frequencies, respectively, and p represents the pitch (electrode spacing + electrode width). In our study, the pitch value is 2 µm (*λ*/2), and the resonant and anti-resonant frequencies are eigenfrequencies we obtainthrough simulation analysis. The equation used to find *k* is as follows:

(3) $$k^{2}=2\;\left(\frac{v-v_{m}}{v}\right)$$

Where *v_m_* is the phase velocity with the metal short, and *v* is the free surface velocity without it.

ZnO, LiNbO_3_, and AlN thin films act as optic and piezoelectric layers in the proposed layer structure as shown in Figure 2a–c, respectively, with IDTs on top. The interaction of light and acoustic waves occurs on the top layer in all the structures. In all of these structures, a SiO_2_ layer is deposited on the silicon (Si) substrate. It is incorporatedbecause the index of refraction of Si is greater than the indexes of refraction of ZnO, LiNbO_3_ and AlN, whereas the index of refraction of SiO_2_ is less than the other materials deposited on top of it. Therefore, the waveguide action cannot be achieved without it [45]. Variation of the electric field in the IDTs, travelling in the top layer of all the structures, produces SAW and penetrates SiO_2_ from the top layer [46]. The light waves fall on the top layer (acting as the optic layer). It creates the acousto-optic effect after interaction with SAW, which is the basic concept of acousto-optic devices [47]. ZnO, LiNbO_3_, and AlN are used in these structures because of their strong piezoelectric and optical characteristics, as well as the ease with which they are deposited [48,49,50,51,52,53,54,55,56]. A material’s *k*^2^ is dependent on characteristics such as dielectric, piezoelectric, and elastic properties. Therefore, the materials are selected based on these characteristics.

Furthermore, while choosing a material for the acousto-optic devices, it should have a suitable acousto-optic figure of merit, which is determined by the photoelastic constant (*p*), density (*ρ*), refractive index (*n*), and acoustic phase velocity (*v*). The acousto-optic figure of merit measures the suitability of a material to modulate the diffraction intensity. The acousto-optic figure of merit (*M*) can be expressed mathematically as:

(4) $$M=\frac{n^{6}p^{2}}{\rho v^{3}}\;$$

All the parameters in Equation (4) should be considered when selecting the top layer material. The refractive index and acoustic velocity are the most essential. The slower the acoustic and optical waves in the material, the more interaction is possible [57]. Some materials offer good optical properties like less losses and high transmittance, but the acousto-optic figure of merit becomes low because of having high acoustic velocity. There are other figures of merit related to acousto-optic devices; however, the acousto-optic figure of merit referred to in Equation (4) is used primarily for gauging the power efficiency of acousto-optic materials. It is preferable to use a combination of materials to achieve a high acousto-optic figure of merit. Therefore, to decrease the overall structure’s velocity and increase the acousto-optic figure of merit, it is better to incorporate the structure with a material possessing low velocity. The velocity of the layer structure is the combined velocity of all the materials. With different combinations of these materials, the acousto-optic figure of merit is calculated.

## 4. Results and Discussion

**Figure 3:**
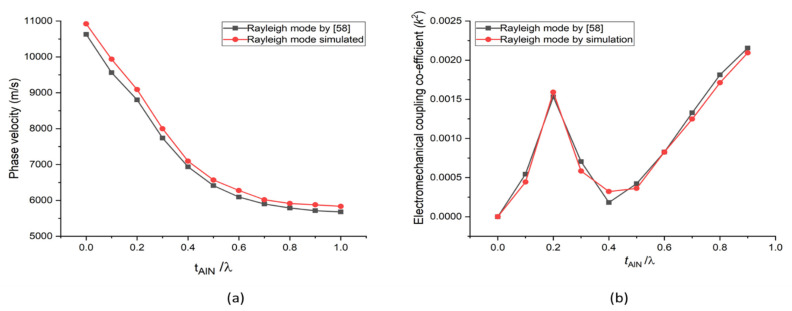
Simulated results of AlN/Diamond structure (**a**) phase velocity (Rayleigh mode) versus normalised thickness of AlN (**b**) *k*^2^ (Rayleigh mode) versus normalised thickness of AlN.

To validate our FEM simulation method used in the structures under study, we first began by simulating the AlN/Diamond (slow–fast) structure, which was previously described [58]. In that study, the propagation characteristics of SAW were theoretically computed using PC acoustic wave software developed by McGill University. SAW propagation in multilayer structures is calculated with this software using the transfer matrix approach, which is implemented in this software. We obtained their numerical data by employing a semi-automated open-source plot digitiser application, i.e., WebPlotDigitizer v. 3.12, which has been utilised in several published papers. To conduct our investigation, we used the unit cell shown in Figure 2 (but with two layers, namely AlN/Diamond) and the boundary conditions shown in Table 2 to simulate the AlN/Diamond structure. The same material constants as those given were utilised [58]. Equations (1)–(3) are used to compute the SAW propagation characteristics. Figure 3 depicts the simulated results, which are somewhat similar to those presented in [58]. The slight inaccuracy can be attributed to the fact that the procedure employed in this case is different from the work presented in [58]. The approach utilised in [58] was based on a transfer matrix method that made assumptions about numerous thin layers’ behaviour to approximate the behaviour of interfacial layers. In contrast, the method employed in this study is based on a finite element method (FEM).

A mesh convergence investigation was also carried out to justify the appropriateness of the chosen mesh density. The mesh density was increased until the SAW velocity values became constant. In this case, the most triangular mesh elements possible were selected (i.e., 120,503). However, increasing the number of mesh elements also increases the processing time. Therefore, to achieve accurate results, it has to be performed at the expense of processing time.

**Figure 4:**
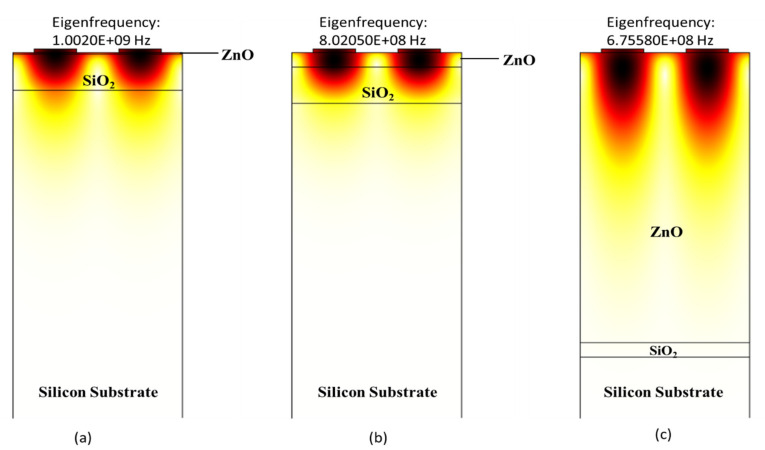
Zoom-in view of wave confinement and displacement profiles of structure A (ZnO/SiO_2_/Si) in Rayleigh mode with the normalised thickness of ZnO (**a**) 0.01, (**b**) 0.1 and (**c**) 2.

**Figure 5:**
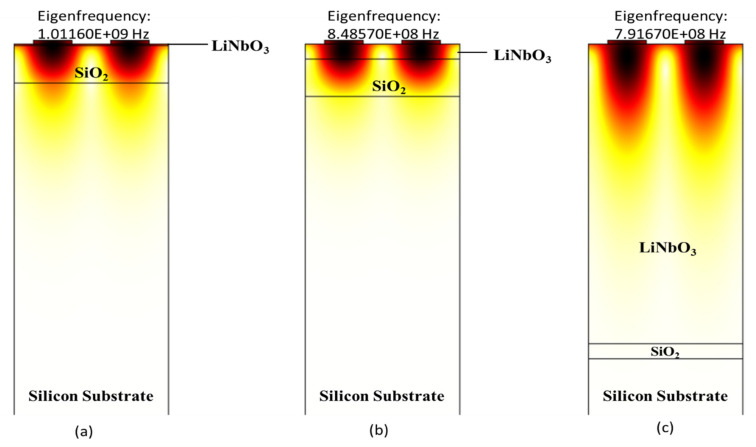
Zoom-in view of wave confinement and displacement profiles of structure B (LiNbO_3_/SiO_2_/Si) in Rayleigh mode with normalised thickness of LiNbO_3_ (**a**) 0.01, (**b**) 0.1 and (**c**) 2.

**Figure 6:**
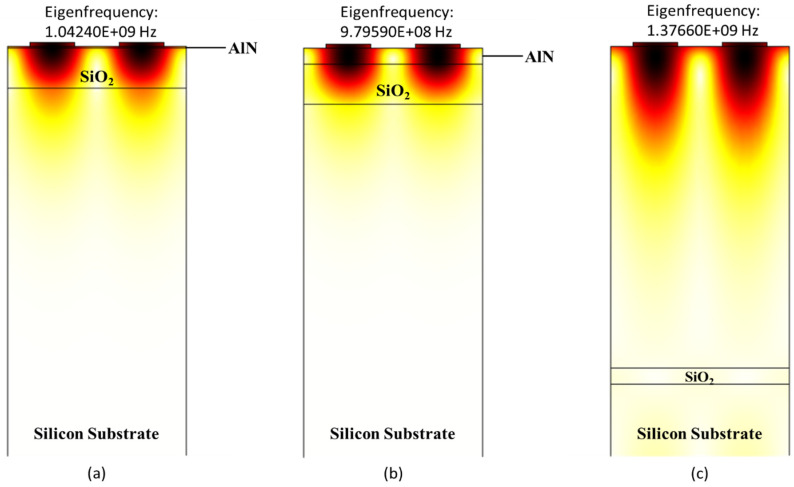
Zoom-in view of wave confinement and displacement profiles of structure C (AlN/SiO_2_/Si) in Rayleigh mode with normalized thickness of AlN (**a**) 0.01, (**b**) 0.1 and (**c**) 2.

In this work, we calculated phase velocity and electromechanical coupling coefficient of structures A (ZnO/SiO_2_/Si), B (LiNbO_3_/SiO_2_/Si), and C (AlN/SiO_2_/Si) on different thicknesses of SiO_2_ (0.4 µm, 1 µm, 2 µm, and 3 µm) with changing thicknesses of ZnO, LiNbO_3_ and AlN (0.4 µm, 0.8 µm, 1.6 µm, 2.4 µm, 3.2 µm and 4 µm) using COMSOL. The values of resonant and anti-resonant frequencies are obtained using COMSOL. Afterward, using Equations (2) and (3) the SAW phase velocity and electromechanical coupling coefficient are calculated, respectively. The polarised mode studied in this work is the Rayleigh mode which occurs in the sagittal plane. The displacement profile for structures A (ZnO/SiO_2_/Si), B (LiNbO_3_/SiO_2_/Si), and C (AlN/SiO_2_/Si) is shown in Figure 4, Figure 5 and Figure 6, respectively.

Figure 4 depicts the displacement profile of structure A (ZnO/SiO_2_/Si). Figure 4a–c shows the displacement profile with the normalised thickness of ZnO as 0.01 λ, 0.1 λ and 2 λ, respectively. It can be vividly seen that by increasing the thickness of ZnO that the surface acoustic waves start to confine in the ZnO layer. It can be seen from Figure 4b that the whole wave is confined in ZnO and SiO_2_ layer and none of it is going in Silicon substrate.

Figure 5 shows the displacement profile of structure B (LiNbO_3_/SiO_2_/Si). The shapes of the mode in the displacement profile help to identify the Rayleigh wave modes. Figure 5a–c shows the results of Eigen frequency analysis for anti-resonance modes at t _LiNbO3_/λ = 0.01, 0.1, and 2 respectively. Moreover, it can be seen clearly from Figure 5a–c that by increasing the thickness of the LiNbO_3_ layer, surface acoustic wave no longer travels in Silicon substrate. Additionally, if it keeps on increasing, it gets entirely confined only in the LiNbO_3_ layer.

Similarly, the displacement profile of structure C (AlN/SiO_2_/Si) is summarised in Figure 6. The displacement profile’s mode shapes help in identifying Rayleigh wave modes. The results in Figure 6a–c were obtained using eigenfrequency analysis in anti-resonance modes for t_AlN_/λ = 0.01, 0.1 and 2. It can be seen that increasing the thickness of AlN thin film value of eigenfrequency increases and the surface acoustic wave no longer passes in Silicon substrate and only travels in AlN and SiO_2_ thin film, as shown in Figure 6b. It continues to do so as the thickness of the AlN layer increases until the entire acoustic wave is contained within the AlN substrate (as seen in Figure 6c). On this point, it reaches a velocity of 5507 m/s at t_AlN_/λ = 2, which is close to AlN’s theoretical acoustic velocity (5600 m/s) [44].

**Figure 7:**
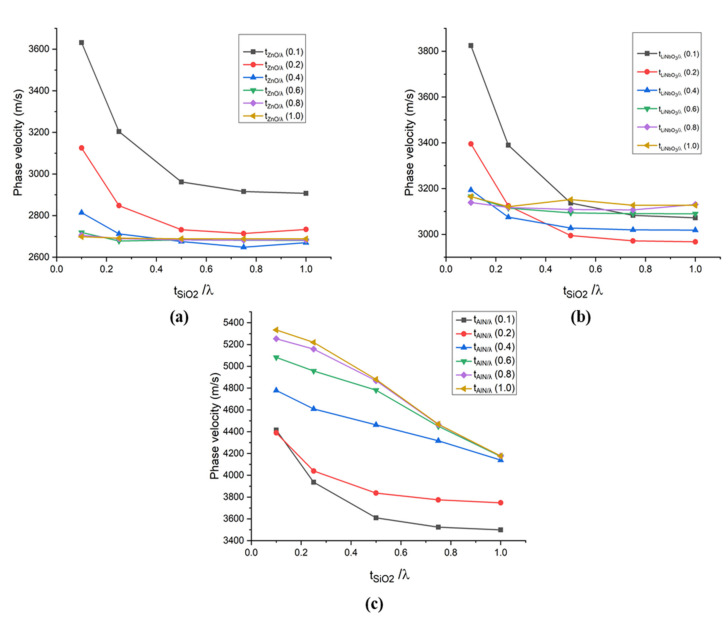
Phase velocity versus normalised thickness of SiO_2_ on different normalised thicknesses of (**a**) ZnO (**b**) LiNbO_3_ (**c**) AlN.

**Figure 8:**
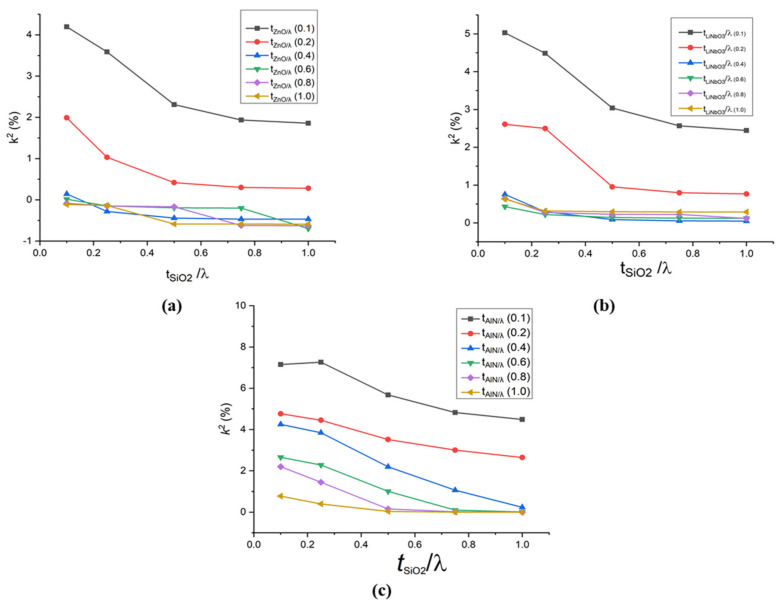
Electromechanical coupling coefficient (*k*^2^) versus normalised thickness of SiO_2_ on different normalised thicknesses of (**a**) ZnO (**b**) LiNbO_3_ (**c**) AlN.

The simulation results for SAW propagation characteristics of the multilayer structures A, B and C are displayed in Figure 7 and Figure 8. In this study, the normalised thickness chosen for SiO_2_ is 0.1 λ, 0.25 λ, 0.5 λ, 0.75 λ and 1 λ. Whereas, 0.1 λ, 0.2 λ, 0.4 λ, 0.6 λ, 0.8 λ and 1.0 λ normalised thickness have been chosen in the case of AlN, ZnO and LiNbO_3_.

It is clear from Figure 7 that the overall phase velocity decreases as the thickness of SiO_2_ increases. All three piezoelectric materials used in structures A, B and C possess high acoustic velocities as compared to SiO_2_. The decrease in the overall behaviour of phase velocity in the structures A, B and C, due to the increase in the normalised thickness of SiO_2_ can be explained using the fact that there is a significant difference between the velocities of SiO_2_ (3750 m/s) with respect to the velocities of AlN (5760 m/s), ZnO (6133 m/s) and LiNbO_3_ (6487 m/s). However, if the change in the normalised thickness of AlN, ZnO and LiNbO_3_ is observed, the case is different. In the case of AlN, when the thickness of AlN increases, the phase velocity also increases compared to the case when the thickness of SiO_2_ was increased. It happens because when the normalised thicknesses of AlN increases, the entire surface acoustic wave travels only in AlN layers, and none of them reaches the substrate. All surface acoustic waves become confined in the piezoelectric layer.

On the contrary, when the thickness of ZnO or LiNbO_3_ increases in structures A and B, as shown in Figure 7a,b, a declined trend is observed. However, by increasing the thickness by more than 0.2 λ, there is no significant change in the behaviour of the phase velocity observed. It happens because the surface acoustic wave becomes confined in the top piezoelectric layer only.

Figure 8 shows the behaviour of the electromechanical coupling coefficient with respect to normalised thicknesses of SiO_2_ and the top layers in the structures A, B and C (i.e., ZnO, LiNbO_3_ and AlN, respectively). The overall electromechanical coupling coefficient behaviour showed a decreasing trend with increased normalised thicknesses of SiO_2_. Figure 8a shows the change in the electromechanical coupling coefficient as ZnO’s thickness increases. It is observed that by increasing the normalised thickness of ZnO, the electromechanical coupling coefficient decreased sharply. However, after the normalised value of 0.4, no significant change in its value is observed. At the smaller values of the normalised thickness of ZnO (t_ZnO_/λ = 0.1 and 0.2), surface acoustic waves are also travelling in SiO_2_. Whereas, when the normalised thickness is increased (greater than t_ZnO_/λ = 0.2), the surface acoustic waves become confined in ZnO, due to which the electromechanical coupling coefficient becomes almost constant. Moreover, in all three structures, the highest value of *k*^2^ is achieved at 0.4 µm thickness of SiO_2_. Additionally, among all three structures, the highest value of electromechanical coupling coefficient is attained in structure AlN/SiO_2_/Si (at 0.4 µm thickness of SiO_2_ and AlN), which is 7.15.

Table 4 illustrates the acousto-optic figure of merit, phase velocity, and electromechanical coupling coefficient of structures A, B, and C. It is evident from Table 4 that among all the structures, structure C (AlN/SiO_2_/Si) exhibits the highest value of electromechanical coupling coefficient (7.15 at the normalised thickness of 0.1 λ of SiO_2_ and AlN layer) with the acousto-optic figure of merit 7.04 × 10^−14^ s^3^/kg. Moreover, it is to be noted that the acousto-optic figure of merit achieved in structure AlN/SiO_2_/Si with the thicknesses as mentioned earlier is higher than the acousto-optic figure of merit of all the individual materials used in these layer structures (i.e., ZnO, LiNbO_3_, AlN and SiO_2_) [29,49].

In light of the results discussed above, it can be concluded that to fabricate an acousto-optic device, structure C (AlN/SiO_2_/Si) would give promising results. However, there is a trade-off between normalised thickness, the acousto-optic figure of merit, acoustic velocity, and electromechanical coupling coefficient. Changing the value of normalised thicknesses, the above said properties could be tailored according to the requirements. Furthermore, AlN is well-suited for use in CMOS technologies since it can be readily deposited by physical vapour deposition at low temperatures, making it a cost-effective option [59,60].

## 5. Conclusions

The study undertaken was targeted towards studying various CMOS compatible multilayer structures that would produce better acousto-optic figures of merit for an AOTF application. SAW propagation characteristics were investigated in three multilayer structures: ZnO/SiO_2_/Si, LiNbO_3_/SiO_2_/Si, and AlN/SiO_2_/Si. It is found that the AlN/SiO_2_/Si multilayer structure gave the highest electromechanical coupling coefficient, i.e., 7.15 at the normalised thickness of 0.1 λ (for SiO_2_ and AlN layer). It also has the highest acousto-optic figure of merit, i.e., 7.04 × 10^−14^ s^3^/kg (which is also higher than the AOFM of individual bulk materials found in literature and mentioned in Table 3). Hence, it is concluded that by using an AlN/SiO2/Si multilayer structure, a high acousto-optic figure of merit can be achieved, which is not possible by using any choice of single bulk material. Moreover, it makes it possible to realise lab-on-chip applications using structure C (AlN/SiO_2_/Si), which is a CMOS compatible SAW structure.

## Figures and Tables

**Table 1:** Dimensions of the device.

Dimensions	Value (µm)
Wavelength	4 (λ)
Pitch of electrode	2 (λ/2)
Width of IDT	1 (λ/4)
Substrate thickness	40 (10 λ)

**Table 2:** Boundary conditions (BC).

Mechanical BC		Electrical BC
Γ1	Free	Zero Charge/Symmetry
Γ2	Continuity	Continuity
Γ3	Continuity	Continuity
Γ	Fixed Constraint	Ground
ΓL, ΓR	Periodic BC	-

**Table 3:** Constants of materials used (AlN, LiNbO_3_, ZnO, SiO_2_ and Si).

Parameter	Symbol	AlN	LiNbO_3_	ZnO	SiO_2_	Si
Density (kg/m^3^)	ρ	3260	4644	5606	2200	2330
Elastic constants (GPa)	c_11_	345	2.03	209.7	78.5	166
	c_12_	125	0.53	12.1	16.1	64
	c_13_	120	0.75	105.4	16.1	64
	c_33_	395	2.45	211.2	78.5	166
	c_44_	118	0.6	42.4	31.2	80
	c_66_	110	-	-	31.2	80
Piezoelectric constants (C/m^2^)	e_15_	−0.48	4.1607	−0.45	-	-
	e_31_	−0.45	0.8661	−0.51		
	e_33_	−1.55	3.7	1.22		
Dielectric constants (10^−11^ F/m)	ε_11_	9	43.6	8.55	3.32	10.62
	ε_33_	11	29.16	10.2	3.32	10.62
Refractive index	N	2.1	2.203	2.015	1.5	3.88
Acoustic velocity (m/s)	*v*	5760	6487.6	6133.5	3750	5000
Acousto-optic figure of merit 10^−15^ [s^3^/kg]	M2	-	10.418	2.572	0.59	-

**Table 4:** AOFM, acoustic phase velocity and *k*^2^ of structures A, B and C (with thickness 0.4 µm for SiO_2_, ZnO, LiNbO_3,_ and AlN layer).

Structure		Figure of Merit (s^3^/kg)	Phase Velocity (m/s)	Electromechanical Coupling Coefficient
**A**	ZnO/SiO_2_/Si	1.23 × 10^−14^	3.63 × 10^3^	4.20
**B**	LiNbO_3_/SiO_2_/Si	5.09 × 10^−14^	3.83 × 10^3^	5.03
**C**	AlN/SiO_2_/Si	7.04 × 10^−14^	4.42 × 10^3^	7.15

## Data Availability

Not applicable.
